# Development and Application of Transcription Terminators for Polyhydroxylkanoates Production in Halophilic *Halomonas bluephagenesis* TD01

**DOI:** 10.3389/fmicb.2022.941306

**Published:** 2022-06-27

**Authors:** Mengmeng Xu, Yue Chang, Yuyan Zhang, Weizhe Wang, Jingyi Hong, Jiping Zhao, Xiaoyun Lu, Dan Tan

**Affiliations:** Key Laboratory of Biomedical Information Engineering of Ministry of Education, School of Life Science and Technology, Xi'an Jiaotong University, Xi'an, China

**Keywords:** *Halomonas bluephagenesis*, intrinsic terminator, termination efficiency, rational design, polyhydroxylkanoates, RNA-Seq, synthetic biology

## Abstract

*Halomonas bluephagenesis* TD01 is one of the ideal chassis for low-cost industrial production based on “Next Generation Industrial Biotechnology,” yet the limited genetically regulatory parts such as transcriptional terminators, which are crucial for tuned regulations on gene expression, have hampered the engineering and applications of the strain. In this study, a series of intrinsic Rho-independent terminators were developed by either genome mining or rational design, and seven of them proved to exhibit higher efficiencies than the canonical strong T7 terminator, among which three terminators displayed high efficiencies over 90%. A preliminary modeling on the sequence-efficiency relationship of the terminators suggested that the poly U sequence regularity, the length and GC content of the stem, and the number and the size of hairpin loops remarkably affected the termination efficiency (TE). The rational and *de novo* designs of novel synthetic terminators based on the sequence-efficiency relationship and the “main contributor” engineering strategy proved to be effective, and fine-tuned polyhydroxylkanoates production was also achieved by the regulation of these native or synthetic terminators with different efficiencies. Furthermore, a perfectly positive correlation between the promoter activity and the TE was revealed in our study. The study enriches our knowledge of transcriptional termination *via* its sequence–strength relationship and enables the precise regulation of gene expression and PHA synthesis by intrinsic terminators, contributing to the extensive applications of *H. bluephagenesis* TD01 in the low-cost production of various chemicals.

## Introduction

*Halomonas bluephagenesis* TD01 is a moderate halophile that can grow under high salt and alkaline conditions (Tan et al., 2011). Open and continuous fermentation process can be conducted based on seawater by *H. bluephagenesis* TD01, making the strain one of the ideal chassis for low-cost industrial production based on “Next Generation Industrial Biotechnology (NGIB) (Chen and Jiang, 2018; Yu et al., 2019a).” *H. bluephagenesis* TD01 is an excellent industrial producer of polyhydroxylkanoates (PHAs), an environmentally friendly biomaterial (Ye et al., 2018). Wild-type *H. bluephagenesis* TD01 accumulates polyhydroxybutyrate (PHB) up to 80% of the cell dry weight, and numerous functional PHAs with excellent properties have also been achieved by engineered *H. bluephagenesis* TD01 (Tan et al., 2011; Meng et al., 2014; Yu et al., 2019b, 2020; Zhang et al., 2022). Tremendous efforts have been made to allow the property improvements of the strain (Tan et al., 2021). Chen et al. completed the whole genome sequencing of *H. bluephagenesis* TD01 (Cai et al., 2011), and several molecular engineering tools such as the conjugation procedure (Fu et al., 2014), expression plasmid (Martinez-Garcia et al., 2020), and the CRISPR/Cas9 gene-editing tool were developed (Qin et al., 2018). Meanwhile, a variety of constitutive and inducible promoters applicable for *H. bluephagenesis* TD01 have been explored in the previous studies (Li et al., 2016b; Zhao et al., 2017; Shen et al., 2018; Ma et al., 2020). For example, the porin promoter derived from a native and highly expressed Porin protein has been proven to be the strongest constitutive promoter in *Halomonas* spp. so far (Li et al., 2016b). Additionally, a powerful T7-like expression system was uncovered for isopropyl-β-D-thiogalactoside (IPTG)-inducible expression of target genes in *Halomonas* spp. (Zhao et al., 2017). Furthermore, a number of ribosome binding sites' (RBS) and other genetic parts have been excavated by means of bioinformatics, while systematic researches are required (Li et al., 2016c). The above molecular tools, synthetic biology approaches, and regulatory parts significantly speed up the engineering of *H. bluephagenesis* TD01 for the low-cost production of various chemicals by NGIB (Tan et al., 2021). Nevertheless, the limited genetically regulatory parts that are critical for the precise control of gene expression and product synthesis have hampered the engineering and applications of the strain. Meanwhile, most of the current regulatory parts for *Halomonas* target the initiation of transcription and translation processes, while few reports are available on the equally influential termination step of transcription.

**Figure 1:**
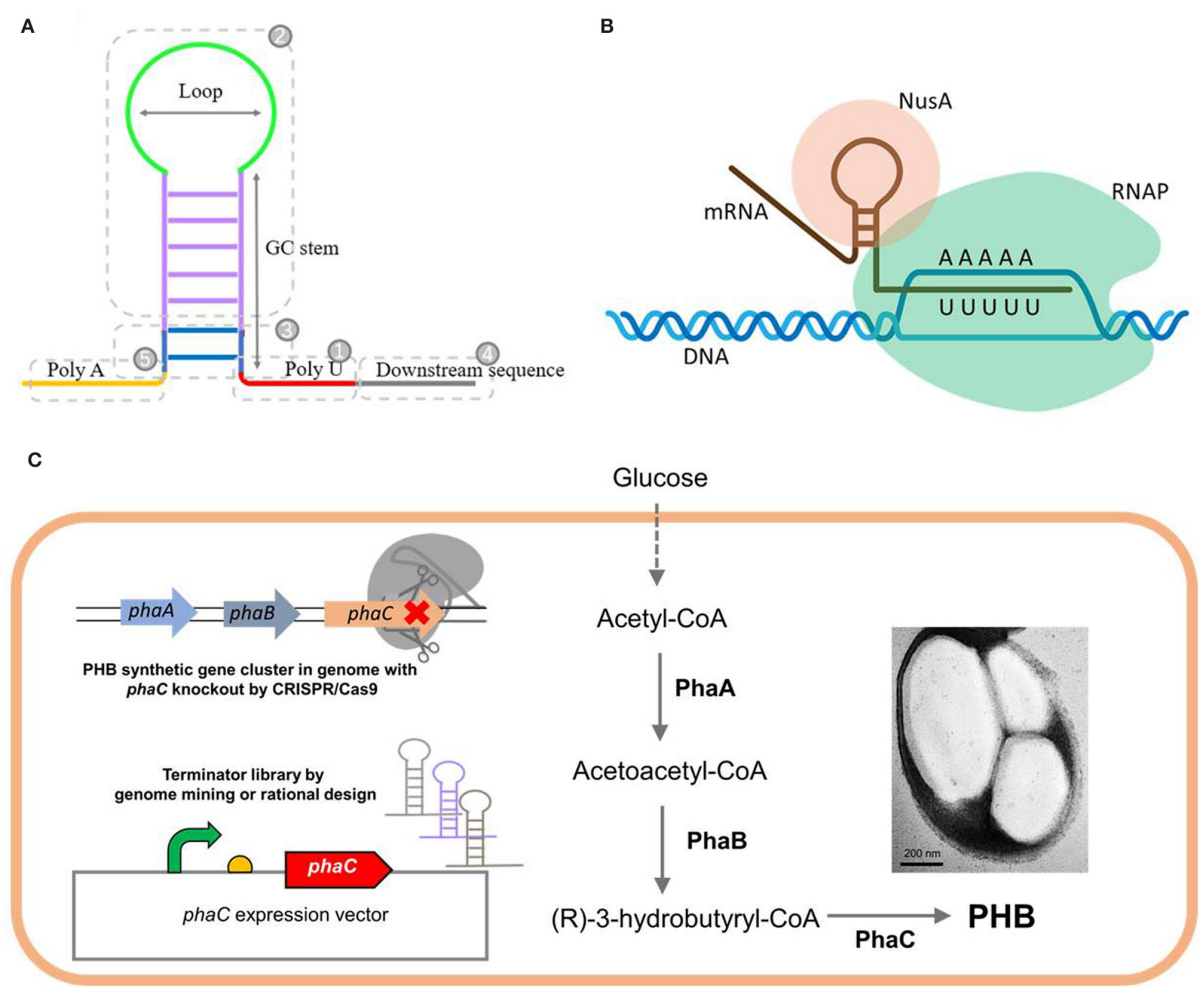
The typical structure **(A)** of an intrinsic terminator and its canonical mechanism for transcription termination **(B)**, as well as the schematic design of this study **(C)**. **(B)** Rho-independent termination does not require any protein factors while it will be enhanced by an accessory proteins NusA. **(C)** The native or engineered terminators were employed to regulate the PHB production, one of the environmentally friendly biomaterials accumulated intracellularly from glucose by three key enzymes: β-ketothiolase (PhaA), acetoacetyl-CoA reductase (PhaB), and PHA synthase (PhaC). The *phaC* gene in the genome of *H. bluephagenesis* TD01 was deleted by CRISPR/Cas9, and functionally rescued by *phaC* expression vectors regulated by terminators with different efficiencies.

About 80% of the transcription termination events in prokaryotes are guided by intrinsic (Rho-independent) terminators, which are located downstream of the gene and show sequence similarity to form a hairpin structure (Santangelo and Artsimovitch, 2011). They typically share a stable GC-rich stem connected by a short loop, flanked by a poly uracil sequence (U tract) downstream and a complementary poly adenine (A tract) sequence upstream (Figure 1A) (Peters et al., 2011; Penno et al., 2015). The termination mechanism of the intrinsic terminator has not been well elucidated. Crucially, a hairpin structure must form within the RNA exit channel of the RNA polymerase (RNAP) (Wilson and von Hippel, 1995; Epshtein et al., 2007). Transcription termination is reported to be initiated when RNAP transcribes and pauses at the end of the U tract, affording sufficient time for nascent mRNA to form a hairpin structure within the exit channel. The transcription complex then disassociates, followed by the release of newly synthesized mRNA (Gusarov and Nudler, 1999; Nudler and Gusarov, 2003; Peters et al., 2011; Ray-Soni et al., 2016). The process does not require the assistance of the Rho factor but will be enhanced by accessory proteins, such as the universal transcription extension factor NusA (Figure 1B) (Greenblatt et al., 1981). Extensive studies on how the hairpin forms and causes the termination are still required.

Transcription termination is an indispensable step to avoid unwanted RNAP read-through, preventing interference between transcript units and recycling RNAP (Ray-Soni et al., 2016). Additionally, it also has been demonstrated to contribute to stabilizing mRNA and enhancing protein expression among prokaryotes and eukaryotes (Curran et al., 2015; Mairhofer et al., 2015; Cheng et al., 2019; Ramakrishnan et al., 2020). Moreover, a weak terminator can be served as a regulatory RNA scaffold to adjust the transcriptional level of genes (Yanofsky, 1981). Collectively, intrinsic terminators are promising to be developed as genetic regulatory parts for gene expression. However, they are currently less well developed in synthetic biology and are mainly limited by the sequence-efficiency relationship of terminators, the basis for their screening and design (Cui et al., 2021).

Intrinsic terminators can be computationally searched in prokaryotic genomes through the identification of hairpins near regions with a high U content (d'Aubenton Carafa et al., 1990; Kingsford et al., 2007; Gardner et al., 2011; Mitra et al., 2011). A highly reliable genome-wide prediction tool for the screening of natural intrinsic terminators in bacteria, RINE, was developed (Gardner et al., 2011). In addition, rational or *de novo* design of synthetic terminators has been performed systematically in *E. coli* (Chen et al., 2013), *Bacillus subtilis* (Cui et al., 2021), yeast (Curran et al., 2015), and mammalian cells (Cheng et al., 2019), offering a set of short terminators that exhibited efficiency levels equivalent to or better than that of native terminators. The predictive sequence features and initial design rules for high efficient terminators in these attempts also facilitate the forward engineering of synthetic terminators (Hudson and Wieden, 2019). Nevertheless, intrinsic terminators often function in a species-dependent manner and exhibit inconstant cross-species performance (Cui et al., 2021).

In this study, we tried to explore the transcriptional intrinsic terminators in *H. bluephagenesis* TD01. Based on the analysis of the transcriptome data of the strain, the potential Rho-independent terminators were mined and characterized using a dual-fluorescent protein reporter system. We also attempted to find the sequence-efficiency relationship by bioinformatics analysis and modeling, which is the basis for the further rational or *de novo* design of novel synthetic terminators. Our native or synthetic terminators were also leveraged to regulate PHB production (Figure 1). Furthermore, the effects of the promoter activity on the termination efficiency (TE) of terminators were investigated in our study. The study enriches the knowledge of transcriptional termination *via* its sequence-strength relationship and provides novel regulatory parts for the precise control of gene expression and product synthesis, accelerating the synthetic biology of *H. bluephagenesis* TD01.

## Materials and Methods

### Oligomers, Plasmids, Bacterial Strains, and Culture Conditions

Bacterial strains, plasmids, and terminators used in this study are listed in Tables 1, 2. Primers used for plasmid construction and validation are collected in Supplementary Table S1. All the oligomers (terminators and primers) were synthesized by BGI Company. A broad-host vector pBBR1MCS-1 (Kovach et al., 1995) was used as the backbone for the construction of the terminator validation plasmids, and a high-copy number vector pSEVA341 (Martinez-Garcia et al., 2020) was employed for PhaC expression in plasmids.

**Table 1:** Bacterial strains and plasmids used in this study.

**Strains/Plasmids**	**Descriptions**	**Sources/ References**
**Strains**		
*E. coli* DH5α	F-*ϕ-5dlacZ*Δlac Δ(*lacZYA-argF*)U169 *deoR recA1 endA1 hsdR17*(*rK*+, *mk*+) *phoA supE44 λ-thi-1 gyrA96 relA1*	Takara
*E. coli* S17-1	*TpR SmR recA, thi, pro, hsdR-M+RP4: 2-Tc:Mu: Km Tn7 λpir*	Simon et al., 1983
*H. bluephagenesis* TD01	*H. bluephagenesis* TD01 wild type, isolated from a salt lake in China	Tan et al., 2011
*H. bluephagenesis* TDΔC	*H. bluephagenesis* TD01 with *phaC* gene deletion	Zhao et al., 2021
*H. bluephagenesis* TD01 (pMCS-P_lib_-sfGFP)	A series of recombinant *H. bluephagenesis* TD01 strains with validation plasmids of different promoters (Porin, H19060, H18740, and ML03285), Cm^R^	Zhao et al., 2021
**Plasmids**		
pBBR1MCS1	A broad host plasmid for *H. bluephagenesis* TD01, Cm^R^	Kovach et al., 1995
pSEVA341	A high-copy-number vector in *H. bluephagenesis* TD01, Cm^R^	Martinez-Garcia et al., 2020
pMCS-P_lib_-sfGFP	pBBR1MCS1 derivative, a series of validation plasmids harboring promoters with different activities (P_lib_: Porin, H19060, H18740, and ML03285), Cm^R^	Zhao et al., 2021
pMCS-Ter-A01–A10, pMCS-Ter-G01–G06, pMCS-Ter-D01–D03	pBBR1MCS1 derivative, a series of terminator validation plasmids with a dual-reporter device of GFP and RFP (Native terminators, A01–A10; Rationally designed terminators, G01–G06; *De novo* designed terminators, D01–D03), Cm^R^	This study
p341-phaC-A01,A02,A04,D01,D02,G04,G05	pSEVA341 derivative, a series of *phaC* expression plasmids with different terminators, Cm^R^	This study
p341-P_lib_-sfGFP-T7-mRFP	pSEVA341 derivative, a series of validation plasmids containing different promoters located upstream of the *sfGFP* gene and a fixed T7 terminator flanked by the *sfGFP* and *mRFP* genes (P_lib_: Porin, H19060, H18740, and ML03285), Cm^R^	This study

**Table 2:** Sequences of all native or designed terminators for *H. bluephagenesis* in this study.

**Terminators**	**Sequence(5^′^ → 3^′^)**	
**Native screened terminators**		
A01	CTTCATGACCTGAAACAAAAGGCGCCCGTGTGGCGCCTTTTGCTTGGTGTGAGGGGT	ON646277
A02	CCCACGTTAAAGAAACGCCGCCCGATTTGGGCGGCGTCTATGTTGGCGCTA	ON646278
A03	GAGACCACTAAAAAAGCAGTGGAAACTGCTAAGCAATAAACGCT	ON646279
A04	AATTGATAGATAAAATGCCGCACCGTCATCATCAGGTGCGGCATTTTTTTGTTTACAC	ON646280
A05	ATCGAATAGAAGCGTTGTGGACGTCAGGCGCCGTTGATATGGCGTCTGACGTTTACGTGGTTGGCTCTTATA	ON646281
A06	TAGAAGCGTTGTGGACGTCAGGCGCCGTTGATATGGCGTCTGACGTTTACGTGGTTGGCTAGCG	ON646282
A07	GCTCTATAAACCAAAGCGCCCGCAAGACCTGCAGAAGGGGCCGCGGGCGCTTTTTTATTGGCGTG	ON646283
A08	TGAACTGATTTAATACGGCGCGTCGTTAGACGCGCCGTTTGCTGGCAGTTAC	ON646284
A09	AACGTTAGATAGAAAGGCGGGCACCAGGAGCGCCCGCCTTTAGAGCCGAAACT	ON646285
A10	TTAGCTAAATACAAAGGCCGGTGGCACTATCTGTGCTACCGGCCTTTTTGTTAAATAGT	ON646286
**Rationally designed terminators based on A04**		
G01	GTGTAAACAAAAAAAGCCGCACCATGACGGTGCGGCTTTTATCTATCAATT	ON646287
G02	GTGTAAATATTGCTTGCCGCACCATGACGGTGCGGCTTTTATCTATCAATT	ON646288
G03	GTGTAAACAAAAAAAGCCGCACCATGACGGTGCGGCTCCACAACTATCAATT	ON646289
G04	AAAAAAAGCCGCACCATGACGGTGCGGCTTTTATC	ON646290
G05	GTGTAAACAAAAAAACTACACCATGACGGTGCTAGTCCACAACTATCAATT	ON646291
G06	GTGTAAACAAAAAAACGCCGCCGCACCATGACGGTGCGGCGGCGTCCACAACTATCAATT	ON646292
***De novo*** **designed terminators**		
D01	AAAAAAAAAAGGCCTCCCGTTAGGGAGGCCTTTTTTTTTT	ON646293
D02	ATCAATAAAAAACGCCGCCCGATTTGGGCGGCGTTATTGTTCGTC	ON646294
D03	GCTATAAAAAAAGGCCGCTTTCGCGGCCTTTTTTCGAAAA	ON646295

*E. coli* DH5α was used for plasmid construction, and *E. coli* S17-1 was a vector donor for plasmid transformation to *H. bluephagenesis* TD01 *via* conjunction (Fu et al., 2014). Wild type *H. bluephagenesis* TD01 (Collection No. CGMCC4353) was used for terminator validation (Tan et al., 2011), and its *phaC* gene knockout mutant *H. bluephagenesis* TDΔC (Zhao et al., 2021) served as another host for PHA production. A series of recombinant *H. bluephagenesis* TD strain harboring validation plasmids of different promoters (pMCS-P_lib_-sfGFP) constructed previously (Zhao et al., 2021) was used to test the activities of different promoters. *E. coli* and *H. bluephagenesis* TD cells were cultured in LB and 60 LB medium (5 g/L yeast extract, 10 g/L tryptone, 60 g/L NaCl), respectively, at 37°C and 200 rpm on a rotary shaker. A total of 25 mg/ml chloramphenicol was added to the medium whenever necessary.

### RNA-Seq Transcriptome Sequencing and Data Analysis

*H. bluephagenesis* TD01 cells were cultured to a density of 1 × 10^7^ cfu. After centrifugation at 4,000 rpm at 4°C, cells were fast-frozen by liquid nitrogen and sent to the Biotree Bioscience Company (Shanghai, China) for total RNA sequencing (Wang et al., 2009). The RNA-seq data were deposited in NCBI with a BioProject ID PRJNA843537. The potential intrinsic terminators were analyzed by TransTermHP software to search the hairpins near the regions with a high U content, and the sequence was further annotated with a 5′ tail, 5′ stem, 3′ stem, and 3′ tail. The free energies Δ*G*_*W*_ for mRNA folding in these terminators were predicted using Mfold (Zuker, 2003). The hairpin structure and the poly U sequence are reported to be the main factors of TE, which were assumed to account for 40 and 60%, respectively in a primary score system (d'Aubenton Carafa et al., 1990). Thus, considering the free energies, the GC content in the stem of hairpin, and the poly U sequence, a total score was assigned to each terminator, and the top 10 native terminators with the highest score were selected for further validation in our study (Supplementary Table S2).

### DNA Manipulations in *E. coli* and *H. bluephagenesis* TD01

A dual-reporter system was constructed for the terminator validation in which different terminators were inserted between two fluorescent proteins, sfGFP and mRFP. The constitutive porin promoter, the *sfGFP* gene, and the *mRFP* gene, as well as the pBBRMCS1 vector backbone were amplified using the primers listed in Supplementary Table S1. The above fragments and the 10 native terminators, synthesized by BGI company, were then assembled *via* Golden Gate assembly (Engler and Marillonnet, 2014) using *Bsa* I restriction sites. The resulting validation Plasmids (pMCS-Ter-A01–A10) contained different terminators flanked by the upstream gene *sfGFP* and the downstream gene *mRFP*. To better characterize the TE, a negative control plasmid (NC) without any termination sequence and a positive control plasmid (PC) with the well-studied strong T7 terminator (Macdonald et al., 1993) between the two reporter genes were also constructed in our study.

For the construction of PhaC expression vector, the porin promoter, the *phaC* gene amplified from *H. bluephagensis* genome, and the selected terminators were assembled into the pSEVA341 vector *via* the Golden Gate method. The expression vectors were further transformed into *H. bluephagensis* TDΔC, a mutant of *H. bluephagensis* with *phaC* gene knockout in the genome by CRISPR/Cas 9 in a previous study (Zhao et al., 2021), to generate recombinant PHA producers TDΔC(A01–G05).

Different promoters with various activities (Porin, H19060, H18740, and ML03285) previously reported (Zhao et al., 2021), were employed to test the effects of promoter activity on the Terminator efficiency. The validation plasmids contained these different promoters which were located upstream of the *sfGFP* gene as well as a fixed T7 terminator flanked by *sfGFP* and *mRFP* genes. The fragments containing different promoters were amplified from previously constructed pMCS–P_lib_-sfGFP (Zhao et al., 2021), and the fragment Containing the T7 terminator and *mRFP* gene was amplified from the aforementioned terminator validation plasmid, followed by the ligation procedure by Golden Gate method to generate a series of plasmids with promoter–terminator pairs (p341-P_lib_-sfGFP-T7-mRFP).

All of the DNA manipulations in this study were based on standard protocols or the instructions of manufacturers. DNA oligonucleotides (Table 2, Supplementary Table S1) were synthesized by BGI Company (Shenzhen, China) and sequenced by Xi'an Tsingke Company (Xi'an, China). Plasmids were first transformed into *E.coli* DH5α and confirmed by colony PCR and sequencing. Then a conjugation procedure was conducted to transform the plasmids from the donor *E. coli* S17-1 to the recipient *H. bluephagensis* TD strains (Fu et al., 2014).

### Determination of Termination Efficiency in *H. bluephagenesis* TD01

*H. bluephagenesis* TD cells harboring the validation plasmids were cultured for 36 h and subjected to an automatic microplate reader (SpectraMax M2e, USA) to test the fluorescence intensities of the GFP and RFP. The excitation wavelength and the emission wavelength of *sf* GFP were set to 485 and 510 nm, respectively, while the excitation wavelength and the emission wavelength of mRFP were set to 584 and 607 nm, respectively. The strain without any plasmid was used as a blank control. The absorbance of cells at 600 nm (OD_600_) was also detected for the normalization of fluorescence intensity.

As terminator sequences were inserted between the GFP and RFP, the Termination efficiency (TE) was determined by the ratio of the fluorescence intensities of GFP and RFP as follows (Cui et al., 2021):


$$\begin{array}{l} TE=\left[1-\left(\frac{RFP}{GFP}\right){\left(\frac{RFP_{0}}{GFP_{0}}\right)}^{-1}\right]\times 100\% \end{array}$$

where TE indicates the Termination efficiency, RFP and GFP are the normalized fluorescence intensities of RFP and GFP respectively in the strain harboring the different terminators, and RFP_0_ and GFP_0_ are the normalized fluorescence intensities of RFP and GFP respectively in the strain harboring the NC plasmid without a terminator between the two fluorescence protein genes.

### Bioinformatic Analysis of the Terminator Structure and Energy and Prediction Modeling

Based on the RNA-seq data, a 60-bp sequence after the stop codon of a gene was selected as the terminator candidate for further analysis. TransTermHP (http://transterm.ccb.jhu.edu/) was used to preliminarily analyze possible terminators in the bacterial genome. The predictions of hairpin structures and their free energies were obtained from Mfold online (http://www.unafold.org/mfold/applications/rna-folding-form-v2.php) using the version RNA Folding Form V2.3 (Zuker, 2003). Considering the kinetic process of RNA folding, the RNA secondary structures and the free energies of characterized terminators were also predicted by Kine Fold from the following website http://kinefold.curie.fr/cgi-bin/form.pl. The settings were as follows: the folding sequences mode: single-strand RNA; the random simulation type: co-transcription folding; the single base addition speed: 20 ms for prokaryotes; and the pseudoknot structures were allowed. The free energy predictions by Kine Fold only focus on the stem-loop sequence without poly A and poly U sequence.

A preliminary modeling of the relationship between the terminator structure and its efficiency were also performed using the Python programming language (Sanner, 1999) and based on the canonical “Thermes model” (d'Aubenton Carafa et al., 1990) and “Hybrid shearing model” (Peters et al., 2011; Santangelo and Artsimovitch, 2011). In our model, Δ*G*_*B*_ (the free energy of the base stacking at the bottom of stem) was obtained by RNAfold Web Server at http://rna.tbi.univie.ac.at/cgi-bin/RNAWebSuite/RNAfold.cgi. The Uscore (the contribution of poly U sequence) and the “match_pattern” (the number of the poly A and poly U pairing) were calculated by Python programming (Supplementary Table S4).

### Production and Characterization of PHB by Recombinant *H. bluephagenesis* TD Strains Harboring Terminators With Different Efficiencies

A modified MMG medium (Tan et al., 2019) was used for the high-yield production of PHB in shake flasks. Briefly, the seed cultures of recombinant *H. bluephagenesis* TDΔC harboring PhaC expression plasmid with different terminators, were grown in a 60-LB medium for 12 h at 37° and 200 rpm on a rotary shaker (IS-RDH1, CTI, USA). Subsequently, the seed cultures in a 5% volume were inoculated into 500 ml of 60-MMG medium and cultivated at 37°C and 200 rpm for 48 h.

Bacterial cells were harvested by centrifugation at 10,000 *g* and washed with distilled water once. Cell dry weights (CDW) were measured after lyophilization, and PHA contents were analyzed using a gas chromatograph (GC-2014, SHIMADZU, Japan) after the methanolysis of lyophilized cells in chloroform, as previously reported (Ma et al., 2020). The PHB granules inside the cells were visualized under transmission electron microscopy (TEM; HITACHI, H-7650B, Japan) based on a previously described protocol (Tan et al., 2014).

### Quantitative Characterization of the Relative Level of Fluorescent Proteins or PhaC by Real-Time qPCR (RT-qPCR)

As previously reported (Fu et al., 2014), the total RNA was isolated from cells, and the cDNA was synthesized using Fastquant RT Kit (Tiangen, Beijing, China) for mRNA analysis. Real-Time PCR assay was performed with SuperReal PreMix (SYBR Green) (Tiangen, Beijing, China) using 16S rRNA as an inner standard. The primers used are listed in Supplementary Table S1.

## Results

### Mining and Characterization of Intrinsic Terminators in *H. bluephagenesis* TD01

**Figure 2:**
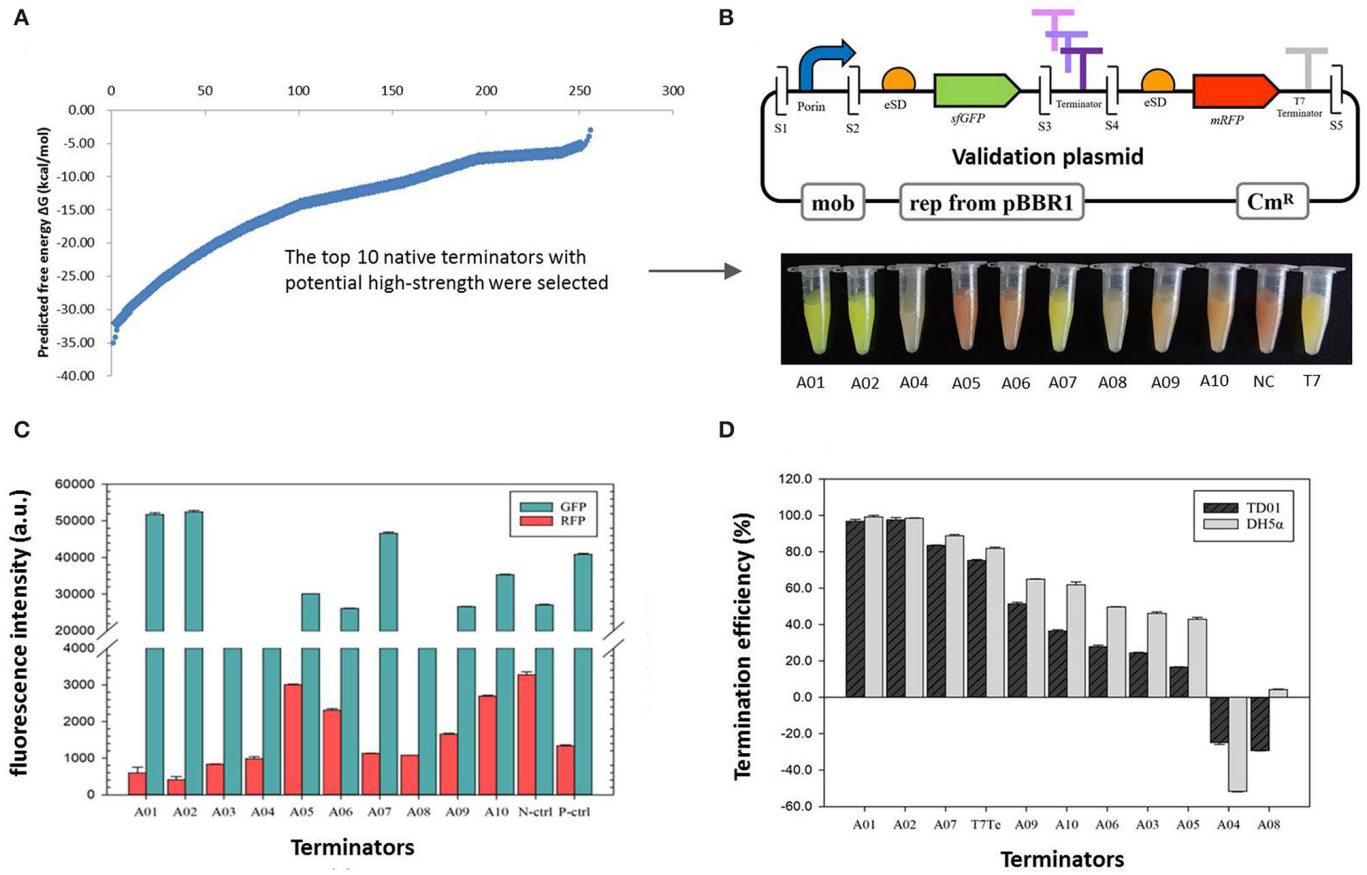
The validation of termination efficiencies of 10 native intrinsic terminators in *H. bluephagenesis* TD01. **(A)** The distribution of the free energies Δ*G*_*W*_ of the 256 native intrinsic terminators mined by RNA-seq data in *H. bluephagenesis* TD01. The top 10 with potential high efficiencies were selected *via* a reported primary rating system. **(B)** The validation plasmids constructed by Golden Gate assembly (up) and the recombinant *H. bluephagenesis* TD strains harboring different terminators showed a variety of colors reflecting different termination efficiencies (down). S1-S5 indicates five *Bsa* I sites. **(C)** The normalized fluorescence intensity of the GFP and RFP in the recombinant *H. bluephagenesis* TD strains harboring the 10 terminators. **(D)** The termination efficiencies of the 10 terminators in both *H. bluephagenesis* TD01 and *E. coli* DH5α. Errors were calculated from three parallel measurements.

In total, 256 potential native intrinsic terminators of *H. bluephagenesis* TD01 were obtained based on the RNA-seq data, with the detailed sequence information of the 5′ tail, 5′ stem, 3′ stem, and 3′ tail analyzed from TransTermHP software (Supplementary Table S5). It is the first collection of computationally predicted intrinsic terminators in *H. bluephagenesis* TD01. The free energies Δ*G*_*W*_ of mRNA folding to form terminators were predicted by Mfold, and the 256 sequences were ranked from the lowest to the highest level according to the value of Δ*G*_*W*_, which was distributed across a wide range from −3.2 to −35.1 kcal/mol (Figure 2A), indicating that the general function of these native terminators might be diverse in *H. bluephagenesis*. The stem-loop structure (hairpin) and poly U sequence are the main contributors to the terminator efficiency, which are assumed to account for 40 and 60%, respectively in a simple rating system according to the literature (d'Aubenton Carafa et al., 1990). Taking the Δ*G*_*W*_, the stem-loop structure, and poly U sequence into our consideration, each terminator was assigned a total score which indicated its potential TE. The top 10 native terminators with the highest score were selected for further validation in our study (Figure 2A).

A set of validation plasmids were successfully constructed by inserting the candidate terminators between two fluorescent protein genes *sfGFP* and *mRFP*, generating pMCS1-Ter-A01–A10. Using this device, the TE was determined by the relative fluorescence intensities of the GFP and RFP. A strong terminator produces less or no RFP, while a weak terminator will produce a high level of RFP (high leakiness). As a result, the cultures of recombinant *H. bluephagenesis* TD01 harboring pMCS1-Ter-A01–A10 showed a variety of colors from green, yellow, and orange to red (Figure 2B), reflecting the different TEs. Inferentially, the terminators A01, A02, A07, and T7 might exhibit higher TEs, while A05, A06, A08, and A04 might have lower TEs.

The fluorescence intensities of the GFP and RFP were further detected by a microplate reader (Figure 2C), and the ratio of the normalized fluorescence intensity was employed to quantitatively characterize the TEs of 10 terminators. The data in Figure 2D and Table 3 showed the TEs ranged from −29.20 to 97.60%, and among them eight sequences displayed termination effects, whereas the remaining A08 and A04 had negative values, indicating no termination functions. Terminators A01, A02, and A07 exhibited higher TEs than that of the canonical T7 terminator, followed by A09, A10, A06, A03, and A05 which demonstrated gradient efficiencies. Terminators A01 and A02 turned out to be remarkably strong terminators with TE values higher than 95%. Thus, our mining provided three native terminators with considerably high efficiencies. Notably, the intrinsic terminators screened from *H. bluephagenesis* TD01 also functioned in *E. coli*, which had slightly higher fluorescent protein expressions and TEs. The result implied that the intrinsic terminators showed consistent trends in the evolutionarily related species and exhibited a high sequence-dependence.

**Table 3:** The main structural features and termination efficiencies of the native or engineered terminators.

**Terminators**	**Loop**	**Stem**	**Poly U/poly A regularity**	**TE**
A01	5 nt, single	6 nt	Perfect regularity	96.9%
A02	5 nt, single	8 nt	Perfect regularity	97.6%
A03	4 nt, single	5 nt	Perfect regularity	24.4%
A04	10 nt, single	8 nt	Perfect regularity	−24.8%
A05	4 nt, single	11 nt	Poor regularity	16.6%
A06	4 nt, single	11 nt	Poor regularity	27.9%
A07	Double loops (5 nt and 9 nt)	11 nt	Perfect regularity	83.6%
A08	4 nt, single	9 nt	Moderate regularity	−29.2%
A09	9 nt, single	10 nt	Moderate regularity	51.4%
A10	5 nt, single	12 nt	Perfect regularity	36.5%
G01	5 nt, single loop (ATGAC)	8 nt	Perfect regularity	50.1%
G02	5 nt, single loop (ATGAC)	8 nt	Poor regularity	39.1%
G03	5 nt, single loop (ATGAC)	8 nt	Poor regularity	32.8%
G04	5 nt, single loop (ATGAC)	8 nt	Perfect regularity, shorter Poly A and Poly U (8 nt each) compared to G01	81.2%
G05	5 nt, single loop (ATGAC)	4 nt	Poor regularity	−33.9%
G06	5 nt, single loop (ATGAC)	12 nt	Poor regularity	−15.7%
D01	4 nt, single loop	8 nt	Perfect regularity	94.7%
D02	5 nt, single loop	8 nt	Poor regularity	88.1%
D03	4 nt, single loop	6 nt	Perfect regularity	89.8%
T7 (Positive control, PC) Macdonald et al., 1993	4 nt, single loop	8 nt	Perfect regularity	75.3%
Negative control (NC)	–	–	–	0

### Determination of the Main Contributors to the Termination Efficiency by Bioinformatics Analysis and Modeling

The relationship between the sequence, the structure, and the efficiency of terminators was preliminary investigated by bioinformatic analysis and modeling in this study. A strong terminator generally has a single stem-loop structure with a loop length of 3–5 nt, a stem length of 5–9 nt, and a perfect poly U sequence (about 8 nt) downstream of the hairpin structure (Peters et al., 2011). The terminators A01, A02, and T7 perfectly displayed the above canonical features and exhibited high-activities, and were used as a reference for our analysis.

**Figure 3:**
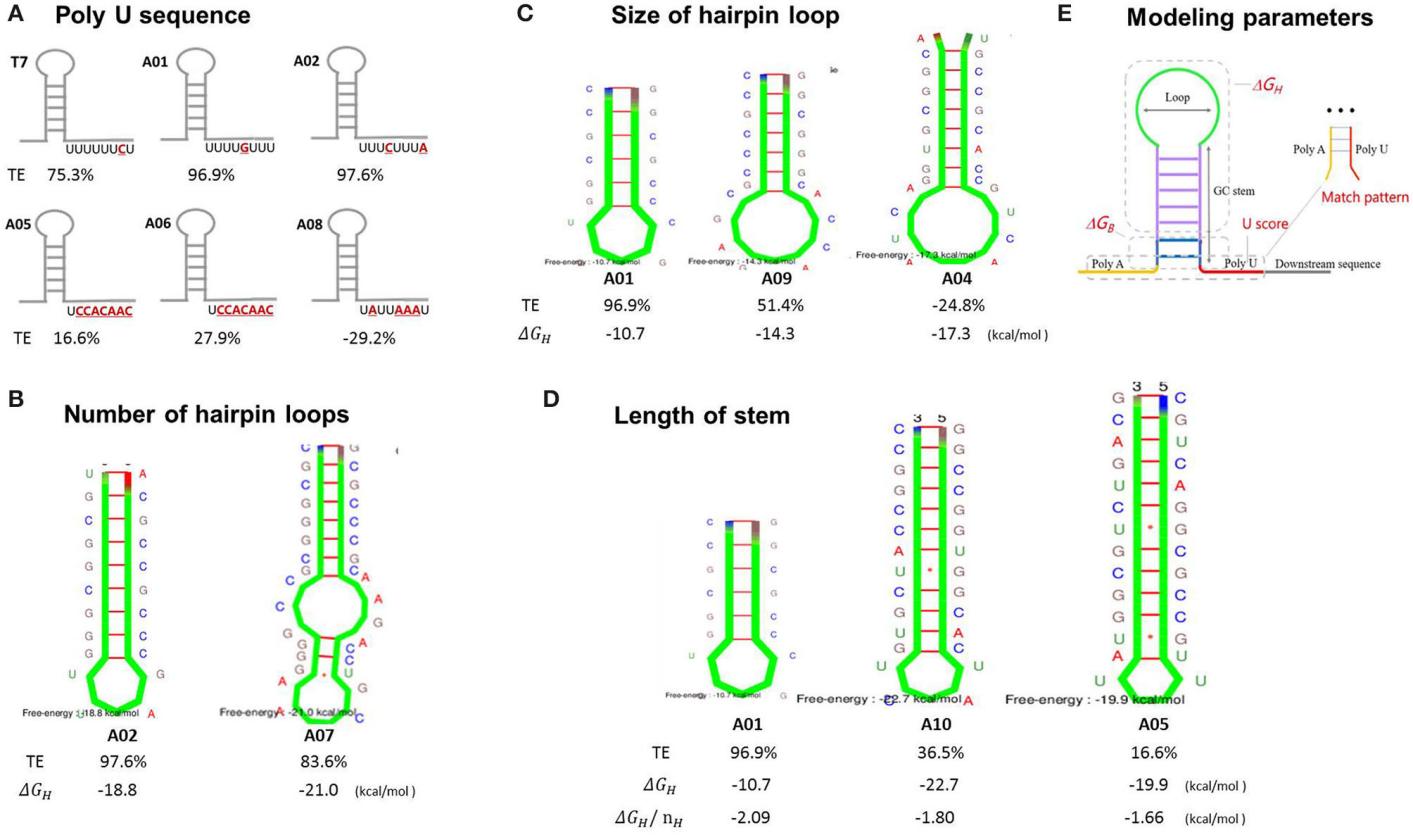
The analysis of the main contributors to the termination efficiencies of terminators. All of the hairpin structures were predicted by Kine Fold using only the stem-loop sequence, without the poly A and poly U sequence nor the upstream and downstream text. The main contributors to termination efficiency were analyzed individually: the contribution of the poly U sequence **(A)**, loop numbers **(B)**, loop size **(C)**, and stem length **(D)**. Taken together, A preliminary model was established to predict termination efficiency based on the parameters indicated in **(E)**. TE, the termination efficiency. Δ*G*_*H*_, the free energy for stem-loop structure formation without the upstream and downstream context. Δ*G*_*H*_/*n*_*H*_, the normalized free energy for the stem-loop structure formation.

The poly U sequence has been recognized to cause a short pause of RNAP, triggering termination events, and is considered to be the most pivotal contributor to TE (Ray-Soni et al., 2016). Terminators A01, A02, and T7 contained a nearly perfect poly U sequence (the ratio of U was super high in the 8 nt poly U sequence), while the terminators A05, A06, and A08 had poly U sequence with poorer regularities (the ratio of U was low), which might be the main reason for the obviously lower TEs (Figure 3A). The formation of the stem-loop structure (hairpin) is also one of the main factors for the termination event. Kine Fold was employed to predict the stem–loop structure of terminators and their free energies Δ*G*_*H*_. As shown in Figure 3B, the terminator A01 and A02 presented a perfect single-loop structure, while the terminator A07 had a unique double-loop structure, leading to a relatively reduced TE, possibly because the low GC content in the stem and a relatively larger size of terminator A07 affected its folding efficiency and stability in the RNAP exit channel. Moreover, the size of the loop may also be one determinant of TE. The terminators A09 and A04 displayed larger loop structures (9- and 10-nt loops, respectively), while lower efficiencies than A01 (5-nt loop) (Figure 3C), as a large loop structure may not be perfectly formed in RNAP exit channel either. Furthermore, the terminator A10 exhibited a lower TE than A01, possibly its increased stem length made it difficult to form a hairpin. This difficulty was reflected by the fact that the normalized free energy required to form the haiprin (Δ*G*_*H*_/*n*_*H*_) increased as the stem length increased (Figure 3D). Terminators A05 or A06 demonstrated lower TEs, as the Δ*G*_*H*_/*n*_*H*_ were even higher than that of A10. Thus, a relatively shorter stem with a higher GC content is thermodynamically favorable to form a stable hairpin. Unexpectedly, the terminator A03 which has a perfect poly U, a small loop, and quite a short stem showed a reduced TE, indicating an excessively small hairpin might cause the fluent read-through of RNAP and a decrease in TE (Supplementary Figure S1). Notably, our results showed that the TE has little relationship with the total free energy of hairpin formation Δ*G*_*H*_ (Figure 3), consistent with the previous findings (d'Aubenton Carafa et al., 1990; Chen et al., 2013).

A preliminary model based on the Python programming language and the previous “Thermes model” and “Hybrid shearing model” was established to predict how the sequence of a terminator affects its efficiency (Figure 3E, Supplementary Figures S3, S4). The “Thermes model” considers the contributions of poly U (U score) and the normalized free energy (Δ*G*_*H*_/*n*_*H*_) (d'Aubenton Carafa et al., 1990), while the “Hybrid shearing model” equates the TE to the probability of RNAP dissociation upon reaching the terminator (Yager and von Hippel, 1991; Peters et al., 2011). Unfortunately, these canonical models turned out to be unable to predict the experimental results of TEs in our study after simulations. Therefore, a customized model was established based on a multiple linear regression equation (Cambray et al., 2013) considering the following main constraints: the normalized free energy of hairpin formation (Δ*G*_*H*_/*n*_*H*_), the free energy of base stacking at the bottom of stem (Δ*G*_*B*_), the poly U sequence (Uscore), and the pairing pattern of poly U and poly A sequence (match_pattern) (Figure 3E). The model was trained by a 582-terminator library reported previously (Chen et al., 2013) and was optimized as follows:


$$\begin{array}{l} TE=4.0458Uscore+2.1789\frac{-\Delta G_{H}}{H}+2.524match\text{\_}pattern\\ +0.2526\Delta G_{B}+55.5843 \end{array}$$

where *TE* is the termination efficiency of terminator; Uscore is the contribution of the poly U sequence; Δ*G*_*H*_ is the predicted free energy of stem-loop structure; *H* is the length of the stem; *match_pattern* is the number of the poly A and poly U pairing; and Δ*G*_*B*_ is the free energy of the base stacking at the bottom of the stem (Figure 3E).

Our modeling proved to be effective to predict the TE when the efficiency was over 50 or 80%, while it was less predictable with the weak terminators (Supplementary Figure S3B), which was quite consistent with a previous study indicating that current models on TE always share the same limitations when predicting weak terminators (Cambray et al., 2013; Cui et al., 2021).

### Characterization of Rationally Designed Terminators

The terminator efficiency can be altered by the “main contributors” engineering strategy based on the terminator A04 with the lowest TE (Table 3). G01–G06 shared a hairpin loop with the same size, which was a relatively stable loop with a 5-nt ATGAC sequence reported previously (Chen et al., 2013). Compared to the terminator A04, G01 only altered the hairpin loop size to 5 nt. Based on G01, the poly A and the poly U sequence in the terminators G02 and G03, respectively, were replaced with ones of poorer regularities; G04 was a truncated G01 with shorter 8-nt poly A and poly U sequences; G05 and G06 contained less perfect poly U sequence and stems with either a reduced GC content or longer length, respectively, which are unfavorable for the hairpin formation.

**Figure 4:**
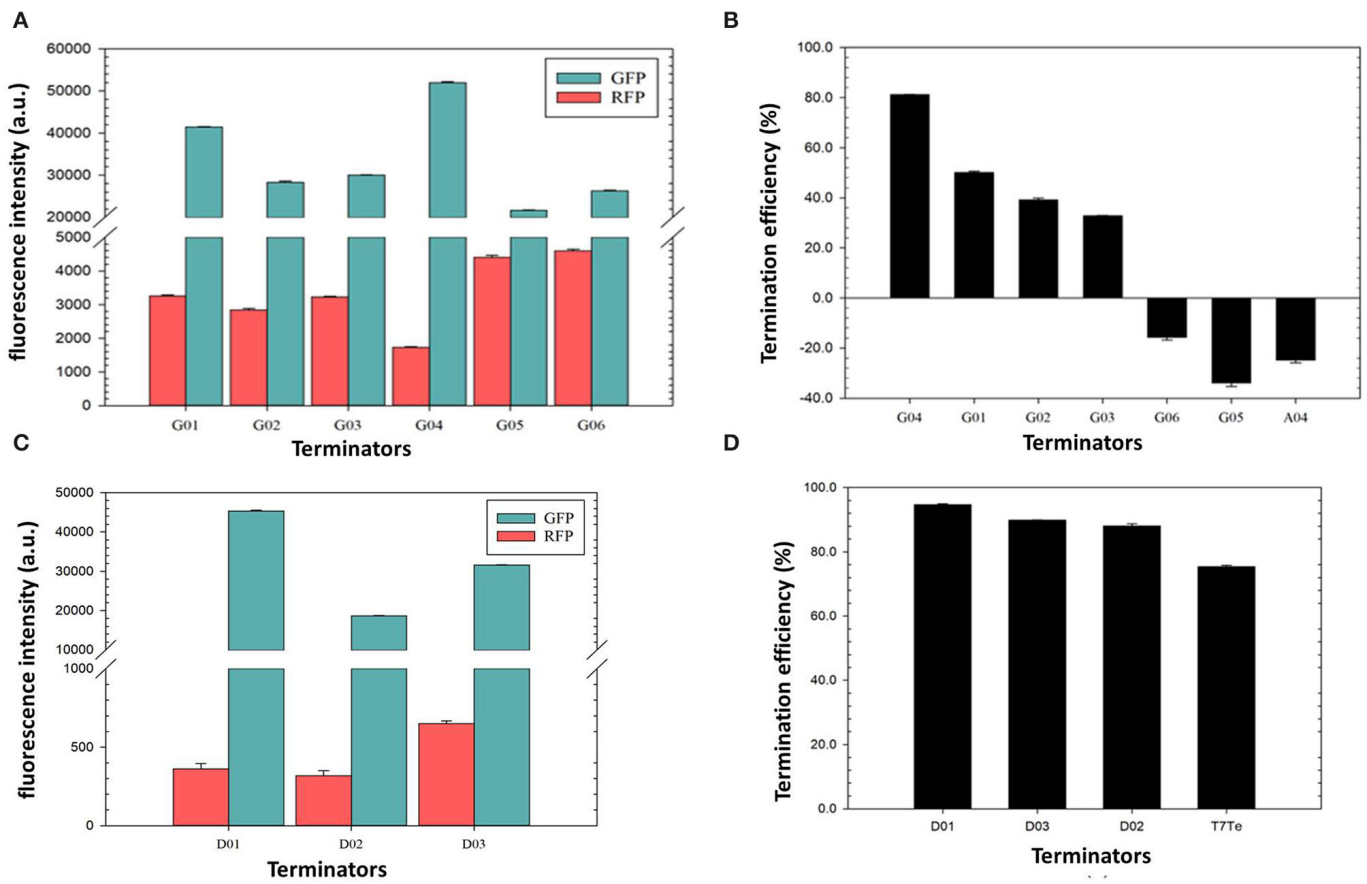
Verification of the termination efficiency of engineered terminators based on the “main contributors” strategy in *H. bluephagenesis* TD01. **(A,C)**: The normalized fluorescence intensities of the GFP and the RFP in the recombinant *H. bluephagenesis* TD strains harboring six rationally designed terminators based on A04 scaffold **(A)** and three *de novo* designed terminators **(C)**. **(B,D)**: The termination efficiencies of rationally designed terminators based on A04 scaffold **(B)** and *de novo* designed terminators **(D)**.

The quantitative characterization by fluorescence intensities of the GFP and RFP showed that the TEs of the novel synthetic terminators, except G05, were higher than that of the original A04 scaffold, which proved that the loop size was the main factor for the low TE of A04 (Figures 4A,B). Among them, the TE of G04 was remarkably higher than that of G01 (81.2 vs. 50.1%), indicating that the flanking sequences of the hairpin structure can be truncated to achieve a higher efficiency. The TE of terminator G02 was lower than that of G01, revealing that a perfect poly A sequence enhanced the TE of the terminator by pairing with the poly U sequence. The TEs of G03, G05, and G06 with poorer poly U regularities were significantly lower than the others, strongly demonstrating that the regularity of poly U sequence is decisive for transcriptional termination. Additionally, the G05 and G06 exhibited negative values of TEs, showing that long stems with low GC content are quite unfavorable for terminator termination, as expected.

### Characterization of *De novo* Designed Terminators

*De novo* design of synthetic terminators was also attempted to increase the flexibility of synthetic biology in *H. bluephagenesis*. According to the criteria for a strong terminator we found, the terminator D01 was designed to contain a perfect 10-nt poly U and poly A sequence, a 8-nt stem with a high GC content, and a relatively stable 4-nt loop with a reported GTTA sequence. The poly U sequence in the terminator D02 was slightly less perfect while it contained a stable 5-nt loop and 8-nt stem with a high GC content. The terminator D03 displayed a perfect poly U sequence, but shorter than the D01 and D02 with a 4-nt loop and a 6-nt stem, expecting that a relatively small hairpin structure will promote termination. The TEs of the three terminators were predicted to be quite high, achieving almost complete termination after modeling and simulation. The quantitative characterization results shown in Figures 4C,D were highly consistent with our predictions. The TEs of three synthetic terminators were higher than 80%, and surprisingly higher than the T7 strong terminator. The less perfect poly U sequence of D02 resulted in a slightly lower TE than D01 and D03 as expected (Figure 3, Table 3). Our attempts showed the good feasibility of *de novo* design based on the sequence-efficiency relationship and “main contributors” strategy, providing several excellent terminators with high efficiencies.

### Determination of the Effects of Promoter Activity on the Terminator Efficiency

**Figure 5:**
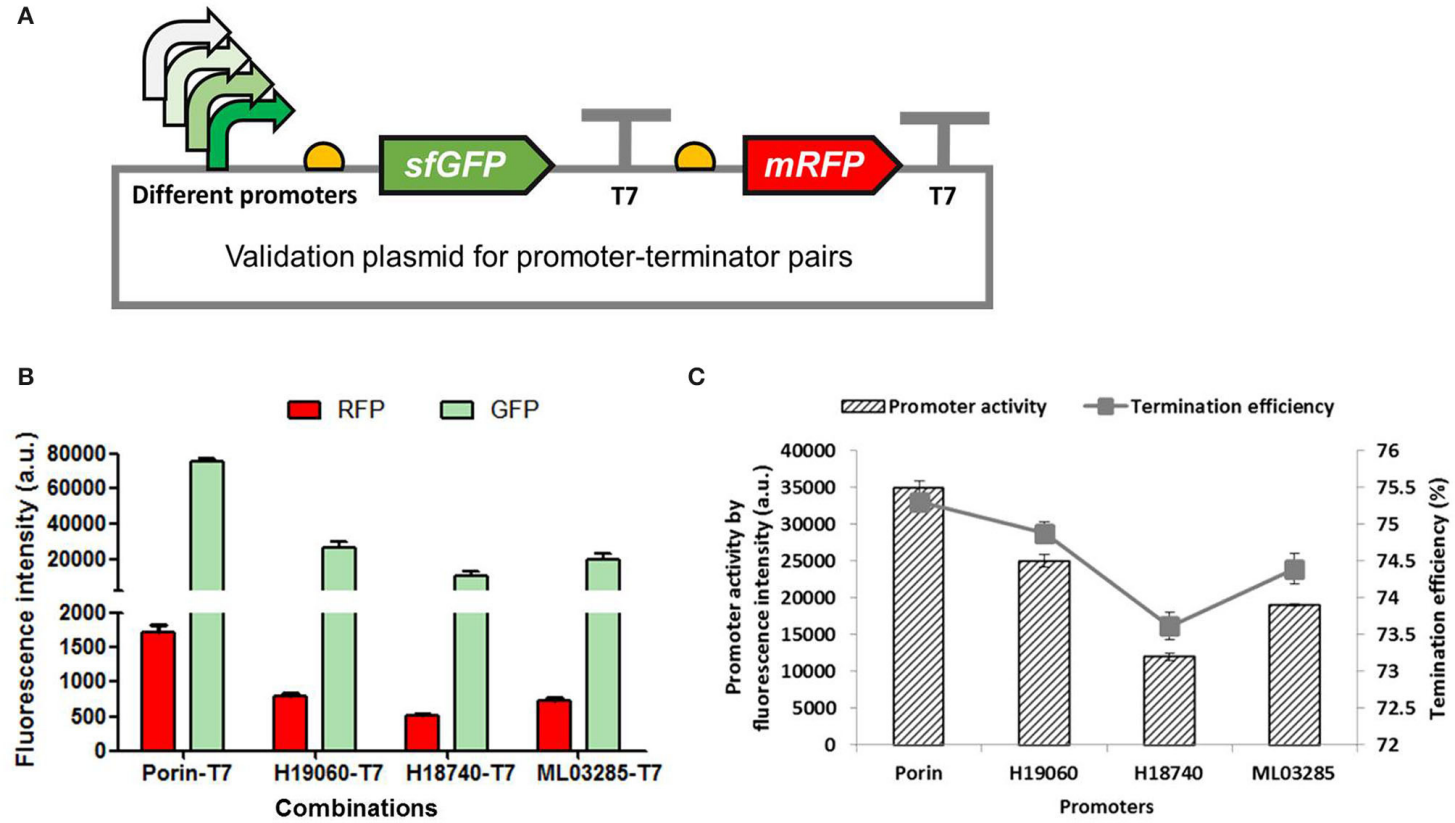
Determination of the effects of promoter activity on the terminator efficiency. **(A)** Validation plasmids harboring promoters with different activities (Porin, H19060, H18740, and ML03285) to promote the expression of *sfGFP* and *mRFP* genes, with the same T7 terminator inserted between the two fluorescent genes. **(B)** The normalized fluorescence intensities of the GFP and the RFP of the recombinant *H. bluephagenesis* TD strains harboring different promoter-T7 terminator pairs. **(C)** The relationship of promoter acitivty and terminator efficiency. The promoter activity was presented by fluorescence intensity of GFP measured in the recombinant *H. bluephagenesis* TD (pMCS-P_lib_-sfGFP) previously constructed.

Promoter regulates the initiation of gene transcription which has been extensively explored as a powerful regulator of gene expression in synthetic biology (Deaner and Alper, 2018). The effect of promoters on the termination of gene transcription was preliminarily investigated in our study. Different promoters with gradient activities (Porin, H19060, H18740, and ML03285) reported previously were employed to regulate the expression of the *sfGFP* and *mRFP* genes, with the same T7 terminator inserted between the two fluorescent genes (Figure 5A). As shown in Figure 5B, the fluorescence intensities of the GFP and RFP shared the same trends with the activities of different promoters, resulting in a perfectly positive correlation between the efficiencies of the promoter and the terminator (Figure 5C), which showed that a strong porin promoter can lead to higher TE of T7 terminator, and the weak H18740 promoter caused a reduced efficiency in the same T7 terminator. The results further demonstrated the complexity of transcription termination as well as the complicated interaction of the whole transcription process.

### PHB Production Regulated by Terminators With Different Efficiencies

**Figure 6:**
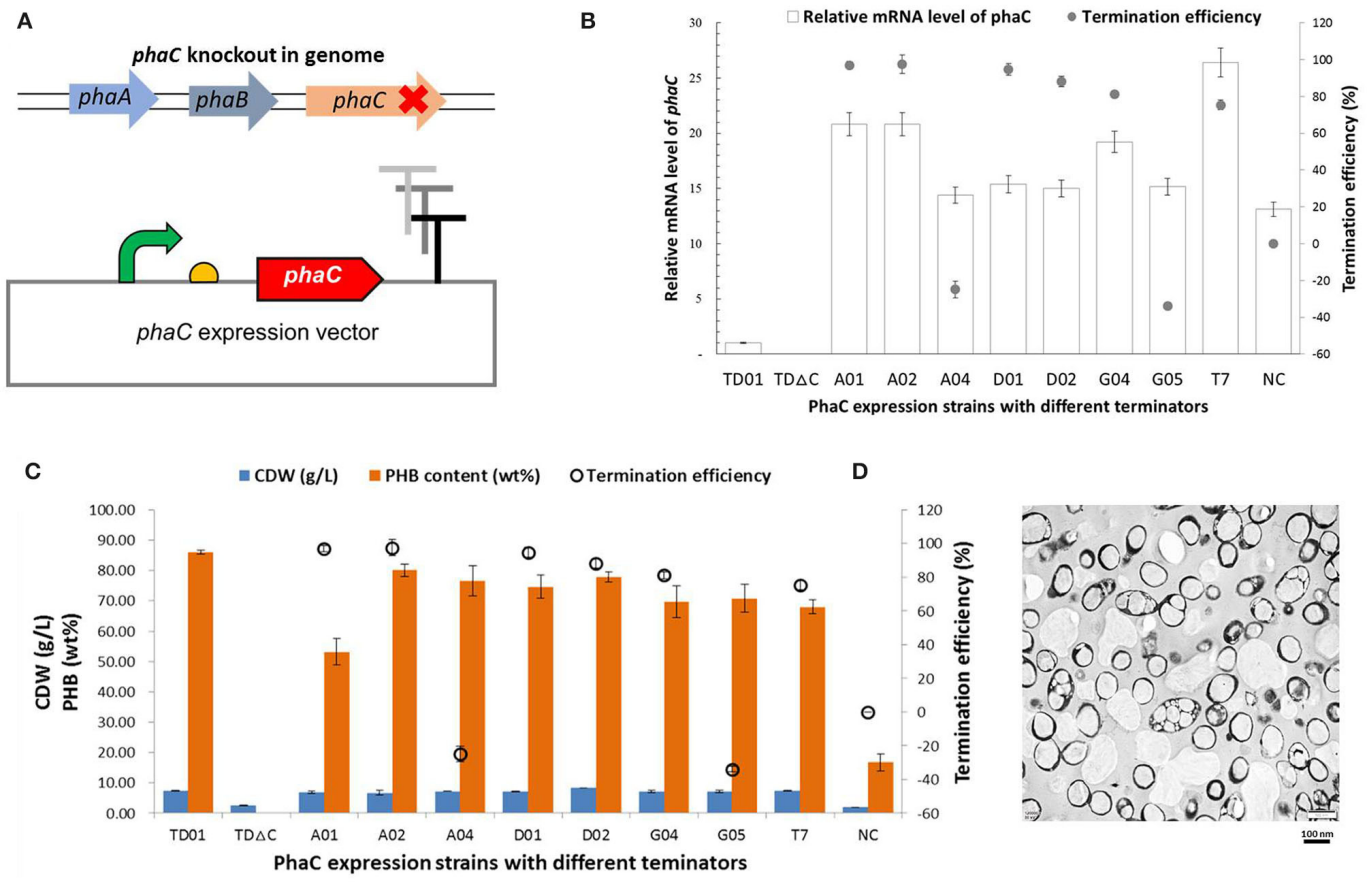
PHB production regulated by terminators with different efficiencies. **(A)** The *phaC* expression vectors were constructed with a series of native or engineered terminators, to rescue the deletion of *phaC* gene in *H. bluephagenesis* TD01 genome. **(B)** The relationship of the relative intracellular mRNA level of *phaC* gene and the termination efficiency. **(C)** Cell dry weight and PHB content in the recombinant *H. bluephagenesis* TD strains harboring different *phaC* expression vectors. **(D)** TEM observations of PHA granules in the recombinant *H. bluephagenesis* TD strains with PHB production regulated by the terminator A02.

Terminators with different efficiencies were further used to regulate the synthesis of the biomaterial PHB, which can be accumulated in high amounts in cells by *phaCAB* genes in the native *H. bluephagenesis* TD01 (Tan et al., 2011). The *phaC* gene in its genome was knocked out by CRISPR/Cas9 and rescued in an expression plasmid with the regulations of different terminators (Figure 6A). The terminators A01, A02, A04, D01, D02, G04, G05, and T7 were selected with efficiencies ranging from −33.9 to 97.6%, and *phaC* gene was expressed without a terminator in the NC group. As shown in Figure 6B, the recombinant *H. bluephagenesis* TDΔC harboring *phaC* expression plasmids exhibited various mRNA levels of *phaC* gene, showing a relatively positive correlation between the TE and mRNA level of *phaC*, in which high levels of mRNA were achieved in case of strong terminators such as A01, A02, G04, and D01. In addition, cell growth and PHB accumulation were determined to be also positively correlated with termination efficiencies to some extent (Figure 6C). The strong terminators A02, D01, and D02 promoted the PHB biosynthesis, with the highest PHB content of 80.12 wt% in the strain harboring terminator A02, as further demonstrated by the TEM observation in Figure 6D. Interestingly, terminators A04 and G05 demonstrated negative TE values and also exhibited considerable mRNA levels of *phaC* and relatively high PHB contents, while the high efficient terminator A01 turned out to accumulate less PHB. These observations implied the complexity of terminator regulation and the effects of metabolic precursors on PHB accumulation. Moreover, the NC group without a terminator downstream *phaC* gene showed remarkably reduced mRNA level, cell dry weight, and PHB content, strongly demonstrating the positive effect of terminator on gene expression and product synthesis. A tuned PhaC expression and PHB accumulation (ranging from 16.75 to 86.12 wt%) were achieved by the regulation of terminators in our study.

## Discussion

*Halomonas bluephagenesis* TD01 is halophilic low-cost industrial producer of various chemicals, yet the limited genetic parts for tuned regulations on gene expression have hampered the engineering and applications of the strain. Promoters and terminators control the key initiation and termination steps of the transcriptional process, which are the focuses of synthetic biology design (Deaner and Alper, 2018). While most of the current regulatory parts for *Halomonas* target promoters, we attempted to enrich the regulatory elements of transcriptional termination for the precise control in *H. bluephagenesis* in this study.

An intrinsic terminator typically contains a short hairpin followed by a poly U sequence, both of which are required for efficient termination of the RNAP flux (Chen et al., 2013). Intrinsic terminators can be easily identified and mined by bioinformatics analysis (Kingsford et al., 2007; Gardner et al., 2011). Their simple structure, small size, and protein independence make them more easily to be redesigned as robust tools for fine gene expression compared to promoters (Cui et al., 2021), which also reduces the possibility of homologous recombination when constructing multiple-gene pathways and circuits (Fujitani et al., 1995; Chen et al., 2013). The termination mechanisms remain largely unknown in detail compared to the well-studied transcriptional initiation. Typically, four steps are involved including the pause of RNAP at poly U sequence, the formation of hairpin in the RNAP exit channel, the disassociation of transcriptional elongation complex, and the release of nascent RNA (Ray-Soni et al., 2016). Three models have been reported to describe the detailed process, the “forward-translocation model” (Santangelo and Roberts, 2004), the “hybrid shearing model” (Peters et al., 2011), and the “allosteric model” (Epshtein et al., 2010). Interestingly, these three models may be not independent and function together. The unclear mechanisms result in the poor relationship between the sequence and the efficiency of terminators, yet mining of native terminators or design of synthetic terminators along with the elucidation of sequence-efficiency relationship is under constant investigation (Hudson and Wieden, 2019).

Strong terminators with high efficiency (TE >90%) can almost completely terminate transcription, while weak terminators with TEs < 50% cause more than half of the polymerase flux to continue beyond the region, and the undesired leakiness is not favored for the tight regulation of genetic circuits in most cases (Chen et al., 2013; Hudson and Wieden, 2019; Cui et al., 2021). Therefore, high efficient synthetic terminators have been developed in *E. coli, Bacillus subtilis*, yeast, and mammalian cells. A library of 582 terminators was established in *E. coli* containing 265 synthetic terminators, yielding 39 strong terminators with Ts >50-fold (over 50-fold reduction in downstream expression, equivalent to TE > 98%). The study also featured four structural contributors to TE: the hairpin loop, the stem base, the A-tract, and the U-tract, and a biophysical model based on a hybrid-shearing mechanism was also developed (Chen et al., 2013). Recently, broad host-range intrinsic terminators adapted for *Bacillus subtilis* and other Gram-positive and Gram-negative bacteria were developed by data-driven and *in silico*-assisted design. A perfect U-tract and hairpin thermodynamics was found to cooperatively contribute to the terminator efficiency. Forward engineering of terminators was performed based on the revealed sequence-activity relationship, and an array of synthetic terminators with efficiencies ranging from 5 to 99% was established (Cui et al., 2021). Meanwhile, synthetic terminators based on a relationship between terminator function and predicted nucleosome positioning of a gene were introduced in eukaryotic *Saccharomyces cerevisiae* to improve the net protein output with increased termination efficiencies up to 96% (Morse et al., 2017). Additionally, a set of 10 endogenous and 30 synthetic terminator variants were evaluated in mammalian cells which held a varying capacity to modulate gene expression through an mRNA half-life based mechanism (Cheng et al., 2019). Our study tried to seek and develop high efficient terminators adapted for *Halomonas* spp. as intrinsic terminators often showed species-dependence in previous cases.

The 256 native terminators were mined with the RNA-seq data and bioinformatics analysis, and the top 10 with potentially high efficiencies were selected upon considering the free energy of whole terminator, the hairpin structure, and the poly U sequence. The TEs of these native terminators ranged from −29.20 to 97.60% (Table 3), providing three native terminators A01, A02, and A07 with considerably higher efficiencies than the strong T7 terminator. However, the TEs of some terminators were found to be negative values such as terminators A04, A08, and the synthetic G05, indicating these sequences might promote the transcription of the downstream genes instead of termination. Combined with the relative mRNA levels of *sfGFP* and *mRFP* genes (Supplementary Figure S2) and the TEs results of the three terminators (Figure 2D, Table 3), it can be speculated that the hairpin might be formed after RNAP read-through, favorably leading to the exposure of the downstream RBS and a subsequent high translation efficiency of the downstream RFP. The phenomenon illustrated that the actual TEs of prokaryotic terminators were the combined effects of transcription and translation, indicating that transcriptional terminators may also regulate the translation process (Li et al., 2016a), and the functions of terminators on gene expressions are challenging to elucidate. Therefore, it is more reasonable to use the protein expression levels (Figure 2C) instead of the mRNA levels (Supplementary Figure S2) of two fluorescent proteins to determine the TEs. However, our results that terminators with a higher TE promoted the expression of GFP and PhaC (Figures 2C, 6B) sufficiently demonstrated that a strong terminator may be beneficial for the expression of upstream genes by enhancing either mRNA stability or translation processes (Curran et al., 2013). Moreover, the 10 intrinsic terminators showed a consistent trend of functions in *H. bluephagenesis* TD01 and in *E. coli*, which is more promising for their use as universal terminators among evolutionarily related species than other reported species-specific terminators (Cui et al., 2021).

To rationally design terminators with desirable efficiency besides direct mining from native hosts, the relationship between the sequence, the structure, and the efficiency of terminators was investigated by bioinformatics analysis and modeling. The findings revealed that the regularity of the poly U and poly A sequence, the length and GC content of the stem, and the number and the size of hairpin loops are the main contributors to TE (Figure 3, Table 3). Generally, a high efficient terminator displays a perfect poly U sequence regularity, a relatively short stem (5–9 nt) with a high GC content, and a single stable loop that is relatively small in size (3–5 nt) (Table 3). Our findings suggest more detailed and predictive determinant features than other studies (Peters et al., 2011). Notably, our results showed that the terminator TE has little relationship with the total free energy of hairpin formation Δ*G*_*H*_, and instead it shows correlations with the normalized free energy (Δ*G*_*H*_/*n*_*H*_), which is consistent with previous findings (Chen et al., 2013). Therefore, the determination of the TEs solely from the ΔG_H_ of hairpin formation or from the Δ*G*_*W*_ of the whole terminator is far from reliable (Supplementary Table S3). That is why the contributions of the poly U regularity and hairpin structure were taken into consideration together with Δ*G*_*W*_ in our first selection of 10 terminator candidates. Multiple biophysical models have been developed to describe how the dominant contributors affect the terminator efficiency (Yager and von Hippel, 1991; Cambray et al., 2013; Chen et al., 2013; Hudson and Wieden, 2019). A preliminary modeling was also performed in our study, which proved to be predictable for the strong terminators, consistent with the previous studies. The poor predictability, especially for weak terminators, is mainly due to the complicated relationship between sequence and efficiency, which may be attributed to the unintelligible mechanisms of the Rho-independent transcriptional termination. With more extensive investigations on the termination mechanisms and the detailed influences of terminators on the translation process, we will be able to accurately determine the sequence-efficiency relationship.

Rational and *de novo* designs of novel synthetic terminators were attempted based on our preliminary analysis of the sequence-efficiency relationship. As a result, the “main contributor” engineering strategy (Table 3) proved to be effective, as the experimental results of synthetic terminator efficiencies were highly consistent with the predictions (Figure 4). Another four novel terminators G04, D01, D02, and D03, exhibiting considerably higher TEs than the strong T7 terminator (Macdonald et al., 1993), were obtained *via* engineering. The criteria that we elucidated for a strong terminator has the potential to be used universally for the mining and design of high-performance terminators in other bacterial strains. The structural determinants for termination and the fundamental principles between terminator sequence and efficiency that we learned from the natural intrinsic terminators, will drive the efficient forward engineering of regulatory terminators. *De novo*-designed terminators failed to achieve the ideal complete termination, which may be attributed to the complexity of the sequence-efficiency relationship and it requires further extensive exploration.

The effect of promoter activity on the termination of gene transcription was preliminarily investigated in our study. Our results showed a perfectly positive correlation between the promoter activity and the TE (Figure 5), indicating that a strong promoter leads to a higher TE and a weak promoter results in the reduced efficiency in the same terminator. A strong promoter generates a high flux of RNAP, which needs to be sharply stopped so as to not interfere with the next transcription unit, thus a strong terminator is evolved. This high start-sharp stop design of cells creates a highly precise pattern of gene expression (Chen et al., 2013). The results further demonstrated the complexity of the whole transcription process in which each step closely interacts with the other. It also suggests a strategy of promoter and terminator engineering combination is favorable for a higher yield of product. Collectively, it can be concluded that the intrinsic terminator-guided transcription termination is highly sequence-dependent, as demonstrated in Figure 3 and Table 3, and also positively correlated with the transcription initiation as shown in Figure 5. Further investigations into the termination mechanism of intrinsic terminators, and their interaction with the other steps of transcription and the translation process, will facilitate the synthetic biology of these genetic regulatory parts.

Finally, the regulation of terminators on the synthesis of the biomaterial PHB was also attempted in our study, and to some extent, the results indicated a positive correlation between the TE and the mRNA level of *phaC* gene as well as PHB accumulation, thus tuned PhaC expression and PHB accumulation were achieved *via* terminator regulation (Figure 6), which will further contribute to the efficient industrial production of PHA by *H. bluephagenesis* TD strain. Moreover, the NC group without a terminator downstream *phaC* gene showed a remarkably reduced cell dry weight and PHB content, also strongly demonstrating the positive regulatory effects of terminators on the gene expression and PHA biosynthesis.

However, the biosynthesis of PHB with different terminators showed mixed results (Figure 6C). The high efficient terminator A01 was unable to produce a high enough PHB content, while terminators A04 and G05 with negative efficiencies resulted in increased PHB contents in cells compared to the NC group. It might be speculated that the flanking sequence of a terminator may also affect its efficiency (Chen et al., 2013; Li et al., 2016a). The TE of a terminator in our study was measured *via* a dual-fluorescent protein device, while its actual TE might vary when it was located downstream of different genes due to the formation of different secondary structures. Meanwhile, complicated communications and interactions of translation and transcription process in prokaryotic cells (Li et al., 2016a) would lead to the varied protein expressions of PhaC upon the same transcript level. Besides the aforementioned reasons for the possibly different PhaC expression levels, the cell growth status, and intracellular metabolic flux or precursors are also critical for PHB accumulation. PHB synthesis is highly growth-related and showed a strong positive correlation with cell dry weight (CDW) (Tan et al., 2011) (Figure 6C). A high cell growth affords a high intracellular metabolic flux, an increased energy and cofactor generation, and a sufficient supply of metabolic precursors like acetyl-CoA for PHA synthesis. Recombinant strains with terminator A01 showed a little lower CDW than others including A04 and G05, which possibly resulted in the reduced metabolic advantages for PHA production. It is notable that *H. bluephagenesis* TD01 wild type functioned better in PHB yield than all of the recombinant strains with synthetic terminators in our study. Recombinant strains were plasmid-born and the addition of antibiotics would somehow inhibit cell growth and PHB accumulation as demonstrated before (Fu et al., 2014). Meanwhile, the sophisticated organization of PHA synthetic gene cluster in the genome of wild-type was also advantageous for PHA biosynthesis. However, the study can be improved by examining the native terminator of genomic *phaC* gene equivalently in plasmid, which will be investigated in the future.

Efficient transcription termination at a defined point is essential for ensuring that different regions of a design do not interfere with each other. As demonstrated in our study, three intrinsic Rho-independent terminators were able to stop transcription with a high efficiency (TE > 90%), avoiding undesired RNAP read-through. The termination efficiencies of native terminators A01, A02, and synthetic terminator D01 were 96.9, 97.6, and 94.7%, respectively, which compared favorably with the reported TE levels in *E. coli* or *B. subtilis* (Chen et al., 2013; Cui et al., 2021). Additionally, the terminator A02 with the highest efficiency remarkably contributed to the PHB production, yielding a high intracellular PHB content of 80.12 wt% (Figures 6C,D), which was also proved that terminators functioned to tune the gene expression and product synthesis. Furthermore, a weak terminator with high leakiness was reported to adjust the transcriptional level of two genes flanked by the terminator (Yanofsky, 1981). Combined with other advantages of terminators such as easily synthesized short length, protein independence, and minimal sequence homology to native sequence (Curran et al., 2015), intrinsic terminators create a novel strategy for fine-tuned gene expression regulation compared to canonical modulation during the initiation of transcription (by promoter) or translation (by ribosome binding sites, RBS). The highly sequence-dependent efficiency of intrinsic terminators makes them more favorable to be designed, engineered, or applied when building functional devices in synthetic biology. However, a more predictable sequence-efficiency relationship and extensive investigations on the termination mechanism are required to facilitate the development of this robust tool.

## Conclusion

*Halomonas bluephagenesis* TD01 is a low-cost industrial producer for various chemicals, and the lack of the genetic regulatory parts has hampered the applications of the strain. This study focused on the transcriptional terminators that are also crucial for the regulation of gene expression, and intrinsic Rho-independent terminators were developed by either genome mining or rational design with high efficiencies of over 90%. A predictable sequence-efficiency relationship was developed by a preliminary model, and the rational and *de novo* designs of novel synthetic terminators were proven to be effective based on the sequence-efficiency relationship and the “main contributor” engineering strategy. The fine-tuned PHB production was also achieved by the regulation of these native or synthetic terminators with different efficiencies. Intrinsic terminators open a new path for the tuned regulations of the gene expression, and our study enriches the knowledge of transcriptional termination *via* its sequence-efficiency relationship and enables the precise regulation of gene expression and PHA synthesis by intrinsic terminators, contributing to the wide applications of *H. bluephagenesis* TD01.

## Data Availability Statement

The data presented in the study are deposited in the NCBI repository with a BioProject ID PRJNA843537 for RNA-seq data and GenBank accession number ON646277–ON646295 for terminators sequences.

## Author Contributions

MX and YC performed the main experiments of terminators design and PHA production, collected and analyzed the data, and drafted the manuscript. YZ and WW participated in the plasmid construction and data collection. JH and JZ performed RNA-seq and analyzed the data. XL and DT supervised the work, revised, and finalized the manuscript. All authors read and approved the final manuscript.

## Funding

This work was financially supported by grants from the Ministry of Science and Technology of China (grant number 2018YFA0900200), National Natural Science Foundation of Shaanxi Province (grant number 2020JQ-027).

## Conflict of Interest

The authors declare that the research was conducted in the absence of any commercial or financial relationships that could be construed as a potential conflict of interest.

## Publisher's Note

All claims expressed in this article are solely those of the authors and do not necessarily represent those of their affiliated organizations, or those of the publisher, the editors and the reviewers. Any product that may be evaluated in this article, or claim that may be made by its manufacturer, is not guaranteed or endorsed by the publisher.
