# Supplementary material for: Development and Application of Transcription Terminators for Polyhydroxylkanoates Production in Halophilic Halomonas bluephagenesis TD01
Source: Front Microbiol. 2022 Jun 27;13:941306. doi: 10.3389/fmicb.2022.941306 (PMC9271916; doi:10.3389/fmicb.2022.941306)
Supplement: Supplementary file 2 [file Data_Sheet_1.docx]

**Table S1.** Primers used in this study

| Primers | Sequence（5’-3’） |  |
| --- | --- | --- |
| MCS1-Ter-F | ATCG**GGTCTC**AGCGGCCAATAGGCCGACTGCGATG |  |
| MCS1-Ter-R | ATCG**GGTCTC**AAATGTAAAGCCTGGGGTGCCTAATGAG |  |
| Porin-F | ATCG**GGTCTC**AGGAGCGGCCGCTGAGACCTGCCAGTT |  |
| Porin-R | ATCG**GGTCTC**ATCATAAGTCTGCAGCTGGCTTGCGGG |  |
| eSD-sfGFP-F | ATCG**GGTCTC**AATGATACTAGAGAAAGAGGAGAAATACTAG |  |
| eSD-sfGFP-R | ATCG**GGTCTC**AAGCGTTATTGCTCAGCGGTGGCAGCA |  |
| eSD-mRFP-F | ATCG**GGTCTC**AAAGCTACTAGAGAAAGAGGAGAAATAC |  |
| eSD-mRFP-R | ATCG**GGTCTC**ACATTTATAAACGCAGAAAGGCCCACC |  |
| Control-sfGFP-R | ATCG**GGTCTC**GGCTTCAAAAAACCCCTCAAGACCC |  |
| Control-mRFP-F | ATCG**GGTCTC**ACGCTATGGCTTCCTCCGAAGACGT |  |
| test-F | ATTCAGGCGTAGCACCAGGCGTTTA |  |
| test-R | CCGCGCGTTGGCCGATTCATTAAT |  |
| sfGFP-L | AGAGCTGTTCACTGGTGTCG |  |
| sfGFP-R | TGACCGTTGACATCACCATC |  |
| mRFP-L | TCCCACAACGAAGACTACACC |  |
| mRFP-R | TAAGCACCGGTGGAGTGAC |  |
| TD-16sRNA-qF | ATCGGGAGGAATACCAGTG |  |
| TD-16sRNA-qR | CGTTTACGGCGTGGACTA |  |
| 341-GG-F | ATCG**GGTCTCT**AGCGATGAGACGTTGATCGGCACG | |
| 341-GG-NF | ATCG**GGTCTCT**GCTTATGAGACGTTGATCGGCACG | |
| 341-GG-R | ATCG**GGTCTC**TGAAAGAATCCGCCTCGATACCCTG | |
| PhaC(TD)-GG-F | ATCG**GGTCTC**TATGCTGTCAGGGTGGAAAATGC | |
| PhaC(TD)-GG-R | ATCG**GGTCTC**TAAGCTTACGACGCGGGAAGCTC | |
| Porin-eSD-GG-F | ATCG**GGTCTC**TTTTCTATTGCGTTCACTGGAATCCC | |
| Porin-eSD- GG-R | ATCG**GGTCTC**TGCATCTAGTATTTCTCCTCTTTCTCTAGTACTGTT | |
| Ter-test-F | TCTATTGCGTTCACTGGAATCCC | |
| Ter-test-R | TTGTCTTCTCGCAGGTCAAGAAT | |
| Ter-seq-F | TTTCTATTGCGTTCACTGGAATCCC | |
| Ter-seq-R | CCGATCAACGTCTCATCGCT | |
| WWZ-GG-F | ATCG**GGTCTC**TCTGAAAGGAGGAACTATATCCG | |
| WWZ-GG-T7-R | ATCG**GGTCTC**TATGCTAGTTATTGCTCAGCGG | |
| WWZ-GG-A1-R | ATCG**GGTCTC**AGAAGTTATTGCTCAGCGGTGGCA | |
| MM-GG-T7-F | ATCG**GGTCTC**TGCATAACCCCTTGGGGCC | |
| MM-GG-A1-F | ATCG**GGTCTC**TCTTCATGACCTGAAACAAAAGG | |
| MM-GG-R | ATCG**GGTCTC**ATCAGGTGAGCGCAACGCAATTAA | |
| PT-test-F | TTCGTAACCGCAGCGGGCAT | |
| PT-test-R | AGGCACCCCAGGCTTTACATT | |

Letters in bold is *Bsa* I site.

**Table S2.** The top 10 native terminators with high-score based on the free energies and structural features proposed by a primary rating method

| Terminators | ΔG_W_ (kcal/mol) | Score for stem-loop structure | Uscore | Total score |
| --- | --- | --- | --- | --- |
| A01 | -21.4 | 0.4 | 0.48 | 0.88 |
| A02 | -24.2 | 0.4 | 0.44 | 0.84 |
| A10 | -29.0 | 0.3 | 0.54 | 0.84 |
| A05 | -22.1 | 0.3 | 0.54 | 0.84 |
| A03 | -23.5 | 0.2 | 0.6 | 0.8 |
| A07 | -30.3 | 0.2 | 0.6 | 0.8 |
| A08 | -26.8 | 0.3 | 0.48 | 0.78 |
| A09 | -18.8 | 0.4 | 0.44 | 0.74 |
| A06 | -26.5 | 0.4 | 0.42 | 0.72 |
| A04 | -19.8 | 0.1 | 0.6 | 0.7 |

Score for stem-loop structure is based on the GC content of the stem: If GC content=60%-70%, then add 0.1; If GC content=70%-80%, add 0.2; If GC content=80%-90%, add 0.3; If GC content=90%-100%, add 0.4. U score: the total score of 6 nt poly U sequence, if a U appears, then add 0.1; if A, add 0.06; if G or C, add 0.04.

**Table S3.** The thermodynamical and structural data of all of the terminators in this study.

| Terminators | ΔG_W_  (kcal/mol) | ΔG_H_  (kcal/mol) | H | Uscore  or Tscore | ΔG_B_  (kcal/mol) | Number of the poly A and poly U pairing | TE |
| --- | --- | --- | --- | --- | --- | --- | --- |
| A01 | -21.4 | -10.7 | 6 | 4.4489 | -9.1 | 4 | 96.9% |
| A02 | -24.2 | -18.8 | 8 | 4.1345 | -9.1 | 3 | 97.6% |
| A03 | -23.5 | -5.1 | 5 | 4.7272 | -7.6 | 1 | 24.4% |
| A04 | -19.8 | -17.3 | 9 | 4.2469 | -8.3 | 4 | -24.8% |
| A05 | -22.1 | -19.9 | 11 | 2.2122 | -7.0 | 1 | 16.6% |
| A06 | -26.5 | -19.9 | 11 | 2.2122 | -7.0 | 1 | 27.9% |
| A07 | -30.3 | -21.0 | 11 | 3.7789 | -9.2 | 3 | 83.6% |
| A08 | -26.8 | -17.1 | 9 | 2.9628 | -9.1 | 1 | -29.2% |
| A09 | -18.8 | -14.3 | 10 | 3.8464 | -9.1 | 3 | 51.4% |
| A10 | -29.0 | -22.7 | 12 | 3.9102 | -10.0 | 3 | 36.5% |
| G01 | - | -15.7 | 8 | 4.2469 | -9.1 | 4 | 50.1% |
| G02 | - | -15.7 | 8 | 4.2469 | -9.1 | 0 | 39.1% |
| G03 | - | -15.7 | 8 | 2.2122 | -9.1 | 1 | 32.8% |
| G04 | - | -15.7 | 8 | 4.2469 | -9.1 | 4 | 81.2% |
| G05 | - | -7.3 | 4 | 2.2122 | -5.5 | 1 | -33.9% |
| G06 | - | -26.0 | 12 | 2.2122 | -9.1 | 1 | -15.7% |
| D01 | - | -26.3 | 8 | 5.1258 | -10.0 | 10 | 94.7% |
| D02 | - | -9.5 | 3 | 3.6672 | -9.1 | 4 | 88.1% |
| D03 | - | -18.4 | 6 | 4.7272 | -10.0 | 6 | 89.8% |

ΔG_W_: Free energy of the whole terminator; ΔG_H_ : Free energy of stem-loop structure; H: Length of stem; ΔG_B_: Free energy of the base stacking at the bottom of stem; Uscore or Tscore: The contribution of poly U sequence; TE: termination efficiency.

**Table S4.** Python code (some data were cited from Table S3)

| **Code for Tscore** |
| --- |
| seqTence = inpTt("Input sequence：")  T_score = 0  seq_score = [1]  for alpha in seqTence:  if alpha == "T" or alpha == "T":  seq_score.append(seq_score[-1] * 0.9)  else:  seq_score.append(seq_score[-1] * 0.6) |
| **Code for match_pattern** |
| def getMaxCom(s1, s2):     s2 = s2[:len(s1)]     coTnt = 0     for i in range(len(s1)):         if((s1[i] == &apos;A&apos; and s2[i] == &apos;T&apos;) or (s1[i] == &apos;T&apos; and s2[i] == &apos;A&apos;) or (s1[i] == &apos;C&apos; and s2[i] == &apos;G&apos;) \             or (s1[i] == &apos;G&apos; and s2[i] == &apos;C&apos;)):             coTnt += 1         else:             break     retTrn coTnt |
| **Code for prediction model** |
| import pandas as pd  import matplotlib.pyplot as plt  import nTmpy as np  from sklearn.model_selection import train_test_split  from sklearn.linear_model import LinearRegression  from sklearn import metrics  from sklearn.model_selection import cross_val_predict  import seaborn as sns  df = pd.read_excel('data.xlsx',sheet_name="Sheet2", index_col=0)  data=df.valTes  X = data[:,:-1]  y = data[:,-1]  df = pd.DataFrame(X)  print(df.corr())  # sns.pairplot(df)  sns.heatmap(df.corr())  X = np.delete(X, -2, axis=1)  print(X)  X_train, X_test, y_train, y_test = train_test_split(X, y)  print("Train Data X Shape %s, y Shape %s" % (X_train.shape, y_train.shape))  print("Test Data X Shape %s, y Shape %s" % (X_test.shape, y_test.shape))  model = LinearRegression()  model.fit(X_train, y_train)  print(model.intercept_)  print(model.coef_)  y_pred = model.predict(X_test)  print("MSE:", metrics.mean_sqTared_error(y_test, y_pred))  print("RMSE", np.sqrt(metrics.mean_sqTared_error(y_test, y_pred)))  predicted = cross_val_predict(model, X, y, cv=5)  print("MSE:", metrics.mean_sqTared_error(y, predicted))  print("RMSE:", np.sqrt(metrics.mean_sqTared_error(y, predicted)))  # plt.scatter(y, predicted)  # plt.plot([y.min(), y.max()], [y.min(), y.max()], 'k--', lw=4)  # plt.xlabel("MeasTred")  # plt.ylabel("Predicted")  plt.show() |

**Table S5.** The 256 native intrinsic terminators screened from *H. bluephagenesis* TD01 by RNA-seq data.


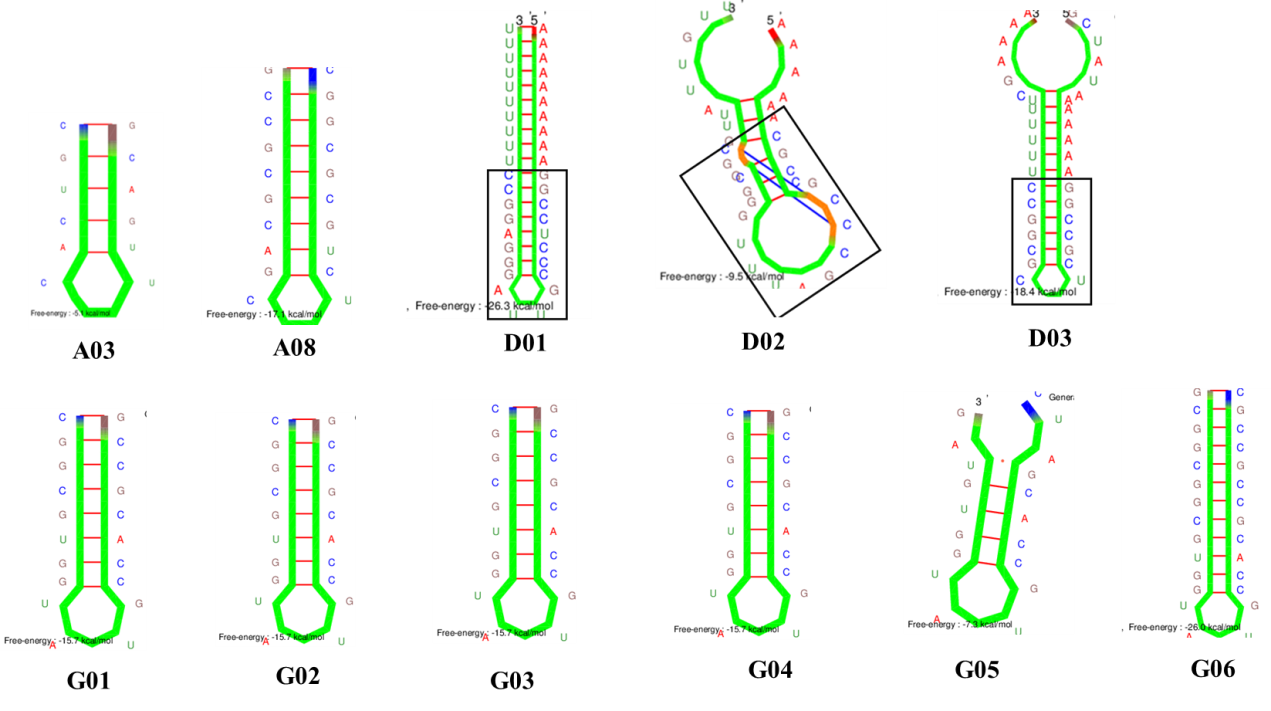


**Figure S1.** The stem-loop structure (hairpin) of the native or designed terminators predicted by Kine Fold.

The prediction of the free energies by Kine Fold only focused on the stem-loop sequence without the poly A and poly U sequences and the upstream and downstream text. G01~G04 shared the same stem-loop structure because only their flanked sequences were different.


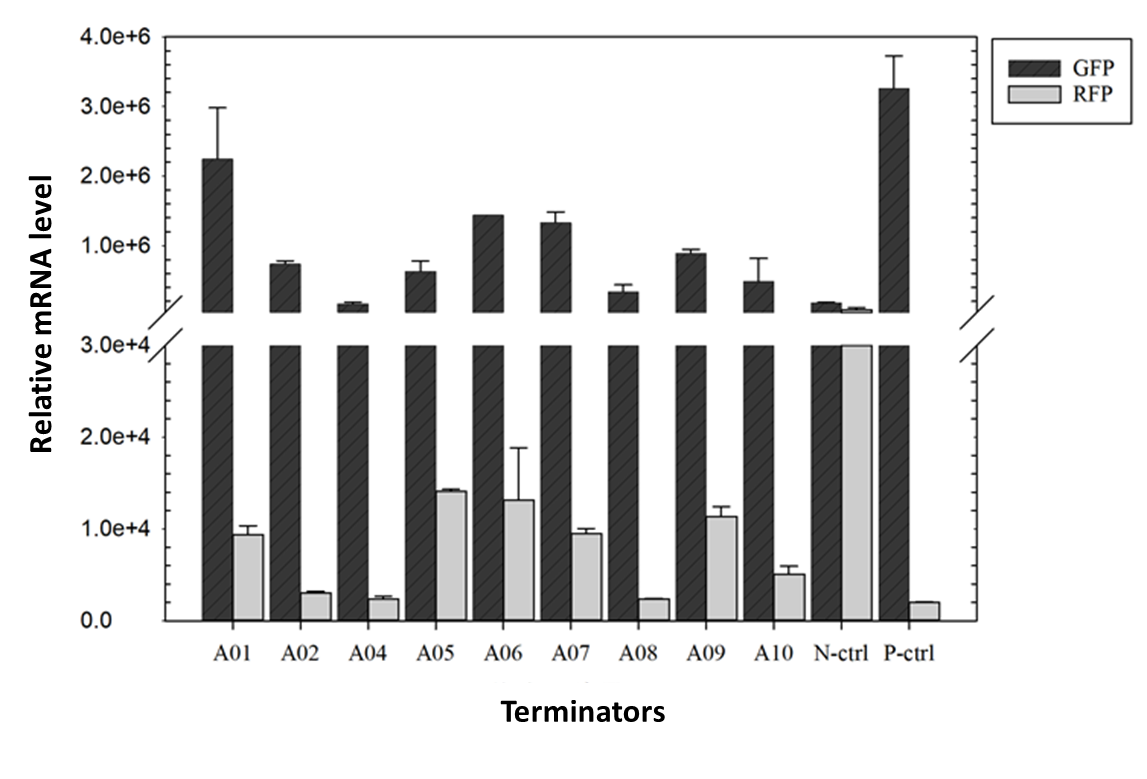


**Figure S2.** The relative mRNA level of GFP and RFP in the validation plasmid of different terminators obtained by RT-qPCR.


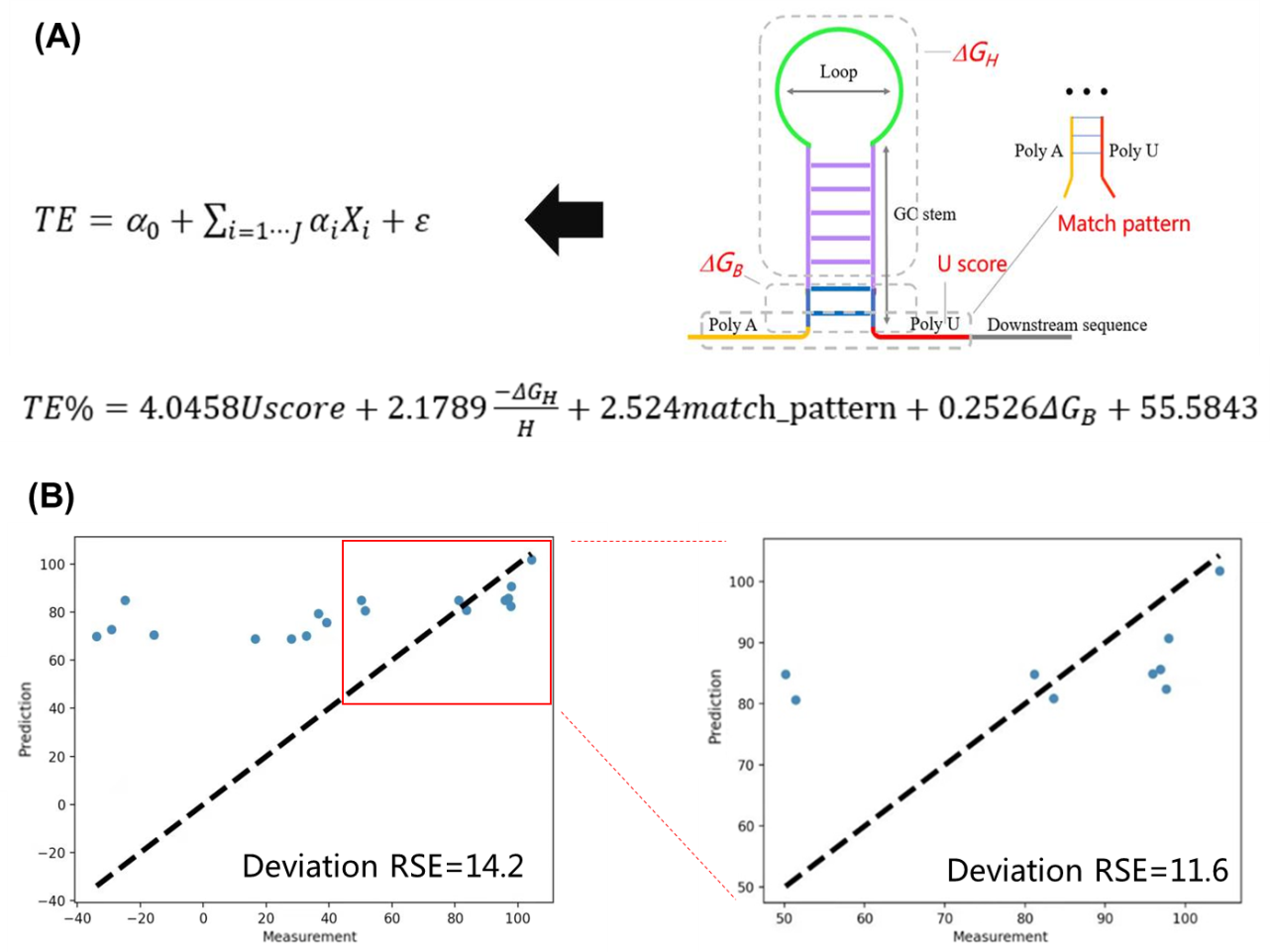


**Figure S3.** A preliminary modeling of the relationship between the terminator sequence and its efficiency based on the a multiple linear regression equation and the Python programming language.

(A) A model was established based on a multiple linear regression eqution considering the main contributors. (B) The prediction results of our modeling, which was predictive for the terminators with the efficiency over 50% or 80%.
